# Restarting anticoagulant therapy after intracranial hemorrhage in patients with atrial fibrillation: A nationwide retrospective cohort study

**DOI:** 10.1016/j.ijcha.2022.101037

**Published:** 2022-04-26

**Authors:** Jong Youn Moon, Gi Hwan Bae, Jaehun Jung, Dong Hoon Shin

**Affiliations:** a Department of Preventive Medicine, Gachon University College of Medicine, Incheon, Republic of Korea; b Artificial Intelligence and Big-data Convergence Center, Gachon University Gil Medical Center, Incheon, Republic of Korea; c Department of Neurology, Gachon University College of Medicine, Incheon, Republic of Korea

**Keywords:** Atrial fibrillation, Intracranial hemorrhage, Warfarin, Direct oral anticoagulant, All-cause mortality

## Abstract

**Background:**

Resuming anticoagulation after an intracranial hemorrhage (ICH) poses a clinical conundrum. The absence of relevant guidelines has led to wide variations in the decision on resuming anticoagulation therapies after ICH. This study aimed to evaluate the risks of an anticoagulation therapy on severe thrombotic events (STE) and severe hemorrhage events (SHE) in Korea and compare the effects of novel direct oral anticoagulants (NOACs) and warfarin in patients with AF.

**Methods:**

This study was performed using the Korean national health insurance claims data obtained between 2002 and 2017 from individuals who had recently survived an ICH with comorbid AF. The endpoints of this study were STE and SHE. Anticoagulants, antiplatelet agents, and non-antithrombotic users were analyzed for survival with propensity score matching.

**Results:**

Among the 4,964 participants analyzed, 878 (17.7%) and 2,070 (41.7%) used anticoagulant and antiplatelet agents, respectively. Anticoagulant (hazard ratio [HR] for STE: 0.385, *P* < 0.0001; HR for SHE: 0.578, *P* < 0.0001) or antiplatelet users (HR for STE: 0.545, *P* < 0.0001; HR for SHE: 0.637, *P* < 0.0001) had a lower risk of STE and SHE than non-antithrombotic users. Anticoagulation 6–8 weeks post-ICH showed a tendency of the lowest risk of all-cause mortality (HR: 0.614, P = 0.0552). However, there was no difference in the risk between the anticoagulant and antiplatelet users. Further, NOACs were associated with a lower risk of STEs than warfarin (HR, 0.263; *P* < 0.0001).

**Conclusions:**

Our results showed that in patients with AF, resuming anticoagulants and antiplatelets after ICH improved the STEs and SHEs. Further, NOAC had additional benefits as compared to warfarin.

## Highlights

• This retrospective cohort study was performed using the Korean national health insurance claims data.
• Compared with non-usage of antithrombotics, a consistent usage of anticoagulants or antiplatelets was associated with lesser severe thrombotic events and severe hemorrhage events in patients with AF who survived an intracranial hemorrhage.
• Furthermore, compared with warfarin, novel direct anticoagulants were associated with a lower risk of thrombotic events.

## 1 Introduction

Atrial fibrillation (AF) increases the stroke risk by 3–5 times and accounts for 15% of the annual cases of cerebral infarction worldwide [1]. Anticoagulant therapy effectively reduces the risk of stroke and systemic embolism in patients with AF [2] and an artificial heart valve [3]. However, because it increases the risk of intracranial hemorrhage (ICH), the risks and benefits of this therapy should be weighed before administration [4], [5]. Post-ICH resumption of anticoagulation in patients with AF for preventing ischemic stroke and systemic thromboembolic events is a longstanding clinical dilemma. No randomized trials have provided reliable estimates of the risk of recurrent ICH, ischemic stroke, or systemic thromboembolism after the resumption of anticoagulants or have investigated whether the benefits of resuming anticoagulants outweigh the risk of recurrent ICH. Therefore, post-ICH anticoagulant usage in patients with AF is based on the clinician’s judgment rather than on evidence-based guidelines [6].

Novel direct anticoagulants (NOACs) were introduced in clinical practice in 2008. These reduce coagulation through direct inhibition of factor Xa (rivaroxaban, apixaban, and edoxaban) or factor IIa (dabigatran); this decreases thrombin formation and fibrinogen conversion to fibrin, thereby reducing clot formation. All NOACs are non-inferior to warfarin in preventing stroke and systemic embolism; moreover, phase III randomized trials have reported that NOACs are associated with a reduced risk of ICH [7], [8], [9], [10]. In a *meta*-analysis of randomized trials on patients with AF, NOACs showed relatively higher efficacy and safety with a significant reduction in the risk of stroke, ICH, and mortality across a wide range of patients [11].

The National Health Insurance (NHI) program in Korea delivers a government-controlled, single-payer, obligatory insurance plan that almost covers the entire Korean population; in 2014, the Korean population comprised approximately 50 million residents [12], [13], [14]. Encrypted customized data tailored for a study protocol can be extracted from the NHI’s raw database with authorization by 1 of the following 2 governmental organizations in Korea: the Health Insurance and Review Assessment (HIRA) and the National Health Insurance Service (NHIS). The research data by the NHIS comprise 6 major files: general information, healthcare services (including in-patient prescriptions), diagnoses, outpatient prescriptions, drug masters, and provider information [12].

Several nationwide observational studies have reported that resumption of anticoagulants in patients with AF and ICH can reduce the mortality relative to that of antiplatelet or non-antithrombotic users [6], [15], [16], [17], [18], [19]. The present population-based retrospective cohort study aimed to evaluate the risks of resuming anticoagulant therapy on severe thrombotic events (STE) and severe hemorrhage events (SHE) in Korean patients with AF who survived an ICH as well as compare the outcomes of anticoagulant and antiplatelet treatments with those of no antithrombotic treatments in such patients. Further, based on the superior safety profile of NOACs relative to that of warfarin, we used the large, nationwide NHIS database to investigate whether treatment with NOACs showed a relatively more improved mortality as compared with warfarin in patients with AF who survived an ICH.

## 2 Methods

The research was conducted ethically in accordance with the World Medical Association Declaration of Helsinki. And this study was reviewed by the Institutional Review Board of the Gachon University Gil Medical Center, which waived the requirement of ethics approval in compliance with the governmental laws and regulations (protocol GFIRB2019-066) and that of informed consent because we only accessed de-identified, previously collected data.

### 2.1 Study population

Customized data of patients diagnosed with an ICH between January 1, 2002 and December 31, 2017 were extracted from the HIRA database; the patients were followed up until December 31, 2017. Diagnoses were coded according to the Korean Standard Classification of Diseases-7, which is based on the International Classification of Diseases, 10th Revision (ICD-10) [12].

Patients with AF who survived an ICH were identified and included according to the following criteria: 1) having an ICD-10 code corresponding to ICH as a diagnosis (I61) and 2) having an ICD-10 code corresponding to AF as a diagnosis (I48) before the onset of ICH. Among the patients with AF who survived an ICH, we excluded those aged < 19 years and those with traumatic ICH, subdural hemorrhage, subarachnoid hemorrhage, structural vascular pathology (e.g. aneurysms and arteriovenous malformation), and tumors [19]. Further, we also excluded patients who received a prescription for both anticoagulant and antiplatelet agents or antithrombotics for < 90 days. To reduce bias due to a severely disabled functional status, we excluded patients who died within 2 months from ICH onset, were hospitalized for more than 80% of the follow-up period, and were not prescribed any drugs after the index ICH.

### 2.2 Variable definitions

The severe thrombotic events (STEs) included ischemic stroke events (fatal or nonfatal), myocardial infarction, or systemic arterial thromboembolism combined with any 1 of the following: emergency room visits, intravenous/intraarterial thrombolysis or thrombus removal, or deaths directly or indirectly caused by a thrombotic event. Severe hemorrhagic events (SHEs) were defined as either recurrent ICH (fatal or nonfatal) or bleeding events directly or indirectly causing death. Supplementary Table 1 lists the ICD-10 codes for the STEs and SHEs. The starting point for outcome follow-up was set at day 60 after the index ICH for a more accurate recording of recurrent events. The starting point for antithrombotic treatment was defined as the time of the first prescription of antithrombotic agents. Mortality was confirmed using the certificate database (recorded data on reasons for changes in the eligibility for NHI or medical aid, death, or emigration).

### 2.3 Anticoagulant and antiplatelet agents

Data on the prescription of anticoagulants and antiplatelet agents were extracted from the claims data obtained within the study period using drug codes based on the Anatomical Therapeutic Chemical Classification. Supplementary Table 2 summarizes the anticoagulant and antiplatelet prescriptions assessed in the present study.

### 2.4 Comorbidities

Comorbidities were defined as additional conditions that developed between 12 months before ICH onset and the end of follow-up. The CHA_2_DS_2_-VASc score was calculated to assess the recurrent thromboembolic stroke risk in patients with AF using a previously reported methodology [6], [20] (Supplementary Tables 3 and 4). The CHA_2_DS_2_-VASc score evaluates the following factors: congestive heart failure, hypertension, age (≥75 years; 2 points), diabetes mellitus, stroke/transient ischemic attack history (2 points), vascular disease, age (65–74 years), and sex (female).

In the present study, the outcomes were STEs and SHEs during the follow-up period. Patients in each cohort were followed from the onset of ICH (entry date) until the occurrence of the study outcome or until December 31, 2017, whichever occurred first.

### 2.5 Statistical analyses

Baseline characteristics were compared according to the drug-use status. Categorical variables were expressed as numbers and percentages, while continuous variables were expressed as means ± standard deviations. The demographic characteristics of the patients were expressed as frequencies and percentages. Three patient groups were compared, namely the anticoagulant, antiplatelet, and non-antithrombotic drug users. The Kaplan–Meier (K–M) curve was used to evaluate the overall survival rates, while the log-rank test was used for group comparisons. To evaluate the independent effects of the prognostic variables, the Cox proportional hazards model was used for multivariate analyses with adjustments for age, sex, and the CHA2DS2-VASc score. A p value < 0.05 indicated statistical significance.

There may be selection bias given the differences in the proportions of basic characteristics among the anticoagulant, antiplatelet, and non-antithrombotic users; this was reasonably addressed through propensity score matching (PSM). The propensity score was defined as the probability of individuals using antithrombotics and was calculated through multiple logistic regression with the following variables: age, sex, factors associated with individual patterns of receiving healthcare service, socioeconomic status (whether participants received medical aid, whether those who received consistent prescription were from the same healthcare facility, and the level of the healthcare facility attended), and specific medical care including emergency room visits and hospitalization within 1 year before entry. We performed three independent propensity score matching with a 1:1 ratio. (Anticoagulant users and antiplatelet usersanticoagulant users and non-antithrombotic users/Antiplatelet users and non-antithrombotic users). We used greedy nearest neighbor matching on the logit of the propensity score.

After PSM, Cox proportional hazards models were repeated to determine the risks and benefits of an anticoagulation therapy for severe thromboembolism and severe hemorrhage as compared with that of an antiplatelet therapy and non-treatment, as well as to determine the optimal time for resuming anticoagulants. In addition, the Cox proportional hazards models were also repeated to compare the effects of warfarin and NOACs on the STEs and SHEs; the results were reported as hazard ratios (HRs) and 95% confidence intervals (CIs). In the Cox models, the age, sex, and CHA2DS2-VASc score were used as covariates, which showed significant differences even after PSM.

All statistical analyses were performed using the SAS version 9.4 (SAS Institute Inc., Cary, NC, USA) and R version 3.5.2 (R Foundation for Statistical Computing, Vienna, Austria) software. Drs. JHJ and DHS had full access to all study data and were responsible for the data integrity and accuracy of data analysis.

## 3 Results

### 3.1 Characteristics of the study population

A total of 375,384 patients were diagnosed with an ICH between January 1, 2002 and December 31, 2017. As shown in the flow chart in Supplementary Fig. 1, we excluded 370,420 patients based on the aforementioned exclusion criteria as follows: age < 19 years (n = 13,305); having a traumatic ICH, subdural hemorrhage, or subarachnoid hemorrhage (n = 59,080); having an AV malformation, brain tumor, or aneurysm (n = 49,626); not having a record of AF before ICH (n = 114,903); prescribed both anticoagulants and antiplatelet agents (n = 5,475); prescribed antithrombotic agents for < 90 days (n = 2,454); hospitalized for greater than 80% of the follow-up period (n = 1,497); and not prescribed drugs after the index ICH (n = 429). Finally, 4,964 patients were included in the present study.

**Fig. 1:**
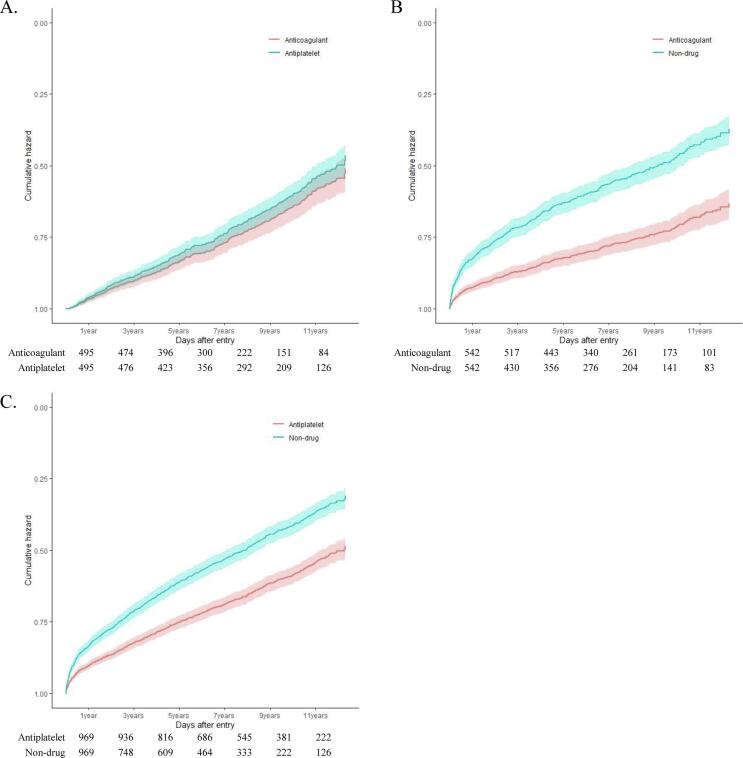
Cox proportional hazards plots for severe thrombotic events related to treatment groups: (A) anticoagulant vs. antiplatelet, (B) anticoagulant vs. non-drug, (C) antiplatelet vs. non-drug.

Table 1 summarizes the baseline characteristics of the study participants. The mean age at entry was 63.83 ± 12.59 years and the mean CHA_2_DS_2_-VASc score was 5.88 ± 1.75. A total of 2,948 (59.4%) patients received prescriptions for antithrombotic agents after entry; among them, 878 (17.7%) and 2,070 (41.7%) were anticoagulant and antiplatelet users, respectively (Table 1). Hypertension, diabetes, and ischemic heart disease were prevalent in 11.9%, 33.2%, and 15.9% of the patients, respectively.

**Table 1:** Demographics of the study population.

	**Before matching**						**After matching**							
	**Total**	**Anticoagulant**	**Antiplatelet**	**Non-drug**	***P***	**Anticoagulant**	**Antiplatelet**	***P***	**Anticoagulant**	**Non-drug**	***P***	**Antiplatelet**	**Non-drug**	***P***
**Total**	4964 (100)	878 (17.7)	2,070 (41.7)	2016 (40.6)		497	497		544 (50)	544 (50)		986	986	
**Age**	63.83 ± 12.59	61.91 ± 11.42	63.77 ± 11.19	62.81 ± 15.17	<0.0001	62.17 ± 10.09	70.03 ± 10.19	<0.0001	61.45 ± 11.26	61.45 ± 11.26		63.21 ± 11.42	63.21 ± 11.42	
**Age group**					<0.0001			0.4947			1.000			1.000
**19**–**29**	61 (1.2)	6 (0.7)	10 (0.5)	45 (2.2)		1 (0.2)	1 (0.2)		3 (0.6)	3 (0.6)		4 (0.4)	4 (0.4)	
**30**–**39**	175 (3.5)	24 (2.7)	47 (2.3)	104 (5.2)		6 (1.2)	6 (1.2)		14 (2.6)	14 (2.6)		24 (2.4)	24 (2.4)	
**40–49**	556 (11.2)	89 (10.1)	192 (9.3)	275 (13.6)		51 (10.3)	51 (10.3)		61 (11.2)	61 (11.2)		107 (10.9)	107 (10.9)	
**50–59**	965 (19.4)	218 (24.8)	384 (18.6)	363 (18.0)		125 (25.2)	125 (25.2)		141 (25.9)	141 (25.9)		198 (20.1)	198 (20.1)	
**60–69**	1572 (31.7)	297 (33.8)	771 (37.3)	504 (25.0)		186 (37.4)	186 (37.4)		186 (34.2)	186 (34.2)		351 (35.6)	351 (35.6)	
**70–79**	1207 (24.3)	207 (23.6)	546 (26.4)	454 (22.5)		117 (23.5)	117 (23.5)		119 (21.9)	119 (21.9)		244 (24.8)	244 (24.8)	
**≥80**	428 (8.6)	37 (4.2)	120 (5.8)	271 (13.4)		11 (2.2)	11 (2.2)		20 (3.7)	20 (3.7)		58 (5.9)	58 (5.9)	
**Sex**					0.0328			1.000			1.000			1.000
**Male**	2730 (55.0)	519 (59.1)	1111 (53.7)	1100 (54.6)		312 (62.8)	312 (62.8)		339 (62.3)	339 (62.3)		571 (57.9)	571 (57.9)	
**Female**	2234 (45.0)	359 (40.9)	959 (46.3)	916 (45.4)		185 (37.2)	185 (37.2)		205 (37.7)	205 (37.7)		415 (42.1)	415 (42.1)	
**Comorbidity**														
**Heart failure**	788 (15.9)	203 (23.1)	352 (17.0)	233 (11.6)	<0.0001	60 (12.1)	50 (10.1)	0.312	60 (11.0)	57 (10.5)	0.769	90 (9.1)	90 (9.1)	1.000
**History of venous Thromboembolism**	89 (1.8)	41 (4.7)	36 (1.7)	12 (0.6)	<0.0001	1 (0.2)	3 (0.6)	0.316	0 (0)	1 (0.2)	0.317	1 (0.1)	4 (0.4)	0.179
**Ischemic heart disease**	907 (18.3)	169 (19.3)	523 (25.3)	215 (10.7)	<0.0001	73 (14.7)	62 (12.5)	0.309	46 (8.5)	45 (8.3)	0.913	92 (9.3)	80 (8.1)	0.338
**Peripheral arterial disease**	863 (17.4)	121 (13.8)	526 (25.4)	216 (10.7)	<0.0001	51 (10.3)	59 (11.9)	0.419	63 (11.6)	40 (7.4)	0.017	85 (8.6)	106 (10.8)	0.110
**Valvular disease**	185 (3.7)	117 (13.3)	32 (1.6)	36 (1.8)	<0.0001	1 (0.2)	1 (0.2)	1.000	7 (1.3)	6 (1.1)	0.780	8 (0.8)	13 (1.3)	0.273
**Hypertension**	588 (11.9)	119 (13.6)	296 (14.3)	173 (8.6)	<0.0001	43 (8.7)	40 (8.1)	0.731	54 (9.9)	43 (7.9)	0.242	75 (7.6)	58 (5.9)	0.127
**Diabetes mellitus**	1648 (33.2)	286 (32.6)	868 (41.9)	494 (24.5)	<0.0001	144 (29.0)	141 (28.4)	0.833	147 (27.0)	133 (24.5)	0.332	243 (24.7)	241 (24.4)	0.917
**Dementia**	565 (11.4)	88 (10.0)	280 (13.5)	197 (9.8)	0.0003	38 (7.7)	33 (6.6)	0.538	31 (5.7)	31 (5.7)	1.000	80 (8.1)	91 (9.2)	0.379
**Hyperlipidemia**	1932 (38.9)	389 (44.3)	1015 (49.0)	528 (26.2)	<0.0001	182 (36.6)	174 (35.0)	0.597	179 (32.9)	181 (33.3)	0.898	295 (29.9)	286 (29.0)	0.657
**CHA2DS2-VASc**	5.88 ± 1.75	5.85 ± 1.73	6.61 ± 1.51	5.18 ± 2.02	<0.0001	5.72 ± 1.78	6.29 ± 1.51	<0.0001	5.62 ± 1.74	5.01 ± 1.92	<0.0001	6.33 ± 1.56	5.20 ± 1.95	<0.0001

CHA2DS2-VASc: congestive heart failure, hypertension, age ≥ 75 years, diabetes mellitus, stroke or transient ischemic attack, vascular disease, age 65–74 years, sex.

### 3.2 STEs and SHEs according to the treatment group

**Fig. 2:**
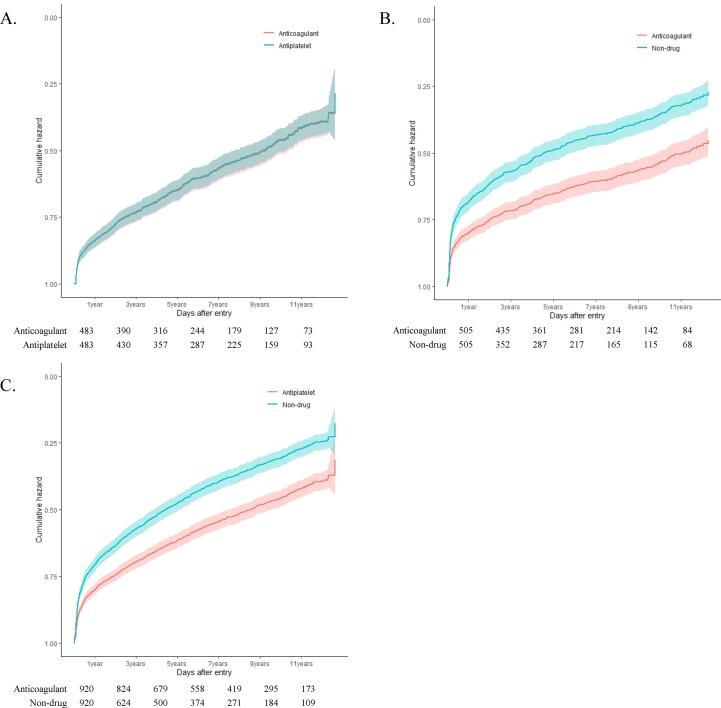
Cox proportional hazards plots for severe hemorrhagic events related to treatment groups: (A) anticoagulant vs. antiplatelet, (B) anticoagulant vs. non-drug, (C) antiplatelet vs. non-drug.

The Cox proportional hazard models of the STEs (Fig. 1) and SHEs (Fig. 2) revealed a significant benefit of anticoagulant and antiplatelet therapy over non-treatment. After adjusting for the age, sex, and CHA_2_DS_2_-VASc scores, anticoagulant users (HR: 0.385, 95% CI: 0.312–0.475; *P* < 0.0001) and antiplatelet users (HR: 0.545, 95% CI: 0.474–0.625; *P* < 0.0001) were noted to have a lower risk of STEs as compared to non-users. However, there was no difference in the risk of STEs between the anticoagulant and antiplatelet users (HR:, 1.168, 95% CI: 0.930–1.465; *P =* 0.181, reference: anticoagulant). Anticoagulant (HR: 0.578, 95% CI: 0.487–0.685; *P* < 0.0001) and antiplatelet users (HR: 0.637, 95% CI: 0.563–0.720; *P* < 0.0001) had a lower risk of SHEs as compared with non-users based on the HRs adjusted for the age, sex, and CHA_2_DS_2_-VASc scores. However, there was no difference in the risk of SHEs between the anticoagulant and antiplatelet users (HR: 1.013, 95% CI: 0.845–1.214; *P* = 0.889) (Supplementary Table 5).

### 3.3 Optimal time for resuming anticoagulant therapy

Resuming anticoagulants between 6 and 8 weeks from the index ICH showed a tendency of a lower risk of all-cause mortality (HR, 0.614; 95% CI, 0.372–1.011; *P* = 0.0552) based on HRs adjusted for age, sex, and CHA2DS2-VASc scores. In addition, resuming anticoagulants between 6 and 8 weeks after ICH onset showed the lowest risk of severe thrombotic events or death (HR, 0.588; 95% CI, 0.362–0.957; *P* = 0.033) while restarting anticoagulants between 4 and 6 weeks after ICH onset showed the highest risk of severe hemorrhagic events (HR, 1.446; 95% CI, 1.12–1.867; *P* = 0.005) based on HRs adjusted for age, sex, and CHA2DS2-VASc score (Supplemental Table 6).

### 3.4 Comparison of the beneficial effects of NOACs and warfarin against STEs and SHEs

**Fig. 3:**
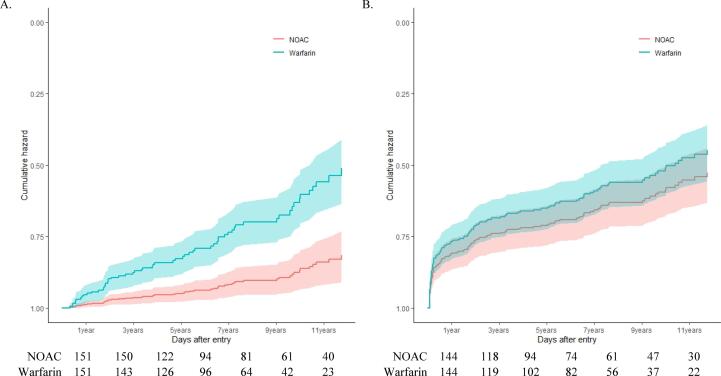
Cox proportional hazards plots for (A) recurrence of ischemic events and (B) recurrence of hemorrhagic events, related to class of anticoagulant.

The survival plots of thrombotic events or death showed that compared with warfarin, NOACs offered a significant benefit in patients with AF who survived an ICH (Fig. 3A). Compared to warfarin, NOACs (HR: 0.263, 95% CI: 0.144–0.480; *P* < 0.0001) were associated with a lower risk of thrombotic events or death, according to the age-, sex-, and CHA_2_DS_2_-VASc score-adjusted HRs (Supplementary Table 6). However, there were no differences in the risk of hemorrhagic events or death between the NOACs and warfarin (Fig. 3B, Supplementary Table 7).

## 4 Discussion

This study showed that compared with non-usage of antithrombotics, a consistent usage of anticoagulants or antiplatelets was associated with lesser STEs and SHEs in patients with AF who survived an ICH. Furthermore, compared with warfarin, NOACs were associated with a lower risk of thrombotic events.

Consistent with our findings, several retrospective studies have reported that compared to non-resumption, resumption of anticoagulants in patients with AF after an ICH significantly reduces the risk of thromboembolism without increasing the ICH recurrence rate. Majeed *et al.* reported that compared with antiplatelet usage, anticoagulant usage caused a greater reduction in the incidence of thromboembolism; however, there were no significant differences in the incidences of mortality and major bleeding between the 2 agents [15]. Nielsen *et al.* reported that anticoagulants significantly decreased the incidences of ischemic stroke and systemic embolism as well as the overall mortality rate [16]. Kuramatsu *et al.* reported that compared with non-treatment, anticoagulants significantly reduced the rates of thromboembolism and mortality without increasing the hemorrhagic complications [17]. Chao *et al.* reported that compared with non-treatment, anticoagulants significantly lowered the incidence of ischemic stroke; however, they were associated with a significantly higher incidence of ICH [18].

In this study, compared with non-treatment, treatment with antiplatelet and anticoagulant agents significantly reduced the STEs without increasing the SHEs. Previous studies have reported that post-ICH antiplatelet medication usage in patients with AF did not reduce the mortality and incidence of thromboembolism and was associated with a similar or increased incidence of SHEs when compared with non-treatment. Nielsen *et al.* reported no differences in the incidences of ischemic cerebral infarction, thromboembolism, and mortality between the non-treatment group and the antiplatelet group; further, antiplatelet agent usage did not increase the recurrence of intracranial and extracranial hemorrhage [16]. Chao *et al.* reported that compared with the non-treatment group, the antiplatelet group showed a similar incidence of ischemic stroke and had a significantly higher incidence of ICH [18]. Pennlert *et al.* showed that compared with the non-treatment group, the antiplatelet group experienced increased thromboembolic events and mortality; however, the incidence of hemorrhagic events did not decrease [19].

The reason for the inconsistency between the findings of the previous observational studies and our findings on antiplatelet usage in survivors of ICH with AF remains unclear. Previous observational studies may have had underpowered sample sizes that could not prove any significant benefit or harm of post-ICH antiplatelet usage in patients with AF. In addition, a significant proportion of patients in the non-treatment groups of the previous studies may have presented with different comorbidities and severities of the sequelae from the ICH, which could have affected the outcomes. The beneficial effects of antiplatelet agents observed in our study could be attributed to nonrandom effects. Finally, the inconsistent findings could also be attributed to between-study differences in the research designs and study participants.

Our study found that the optimal timing of restarting anticoagulants may range between 6 and 8 weeks from the onset of ICH. Specifically, resuming anticoagulation between 6 and 8 weeks showed a tendency of lower all-cause mortality compared with other resumption times. Additionally, the aforementioned resumption time had a significantly lower reduction of severe thrombotic events. Further, anticoagulation resumption between 4 and 6 weeks from the index ICH had a significantly higher risk of severe hemorrhagic events compared with other periods. Majeed et al. analyzed 234 patients with ICH using warfarin and found that using anticoagulants after 10–30 weeks had the lowest incidence of combined ICH and ischemic cerebral infarction [15]. Hawryluk et al. performed a systematic review of extracted data on hemorrhagic and thromboembolic events following the index ICH and found that restarting anticoagulants after 72 h was significantly associated with thromboembolic complications while restarting before 72 h increased the hemorrhage risk [21]. Pennlert et al. reported that anticoagulant usage significantly reduced the incidence of thromboembolism at initiation between 4 and 16 weeks without increasing the incidence of hemorrhagic complications. Moreover, the incidence of vascular death and stroke was lowest at initiation between 7 and 8 weeks in the analysis of the Swedish Stroke Registry [19]. These previous findings are consistent with our result that resuming anticoagulants between 6 and 8 weeks could be the optimal timing.

In this study, compared with warfarin, NOACs were associated with lesser thrombotic events. Randomized trials and several systemic reviews on NOACs and real-world data analysis in patients with AF have shown that NOACs are non-inferior to warfarin in the prevention of ischemic stroke and severe systemic embolism. Moreover, they are associated with a lower risk of severe or intracranial hemorrhage [7], [8], [9], [10], [11]. An observational study by Nielsen et al. reported an association of NOACs, but not warfarin, with a non-significantly lower risk of ischemic stroke and recurrent ICH [22]. Because routine monitoring and dose adjustment of warfarin is difficult in disabled patients after ICH, the time in therapeutic range in patients treated with warfarin may be suboptimal. This can partially explain the lesser thrombotic events associated with NOACs than with warfarin. There are multiple randomized trials currently assessing the hypothesis that NOACs are non-inferior in preventing ischemic stroke and STEs, and superior in reducing SHEs and mortality, to antiplatelet or non-thrombotic agents in patients with AF and ICH, as well as the optimal time of resuming anticoagulants [23], [24]. Therefore, our results suggest that the findings of the ongoing trials will be positive.

This study has some limitations. First, the customized HIRA data lack information on the laboratory tests results, lifestyle, or family history, which may be associated with ischemic stroke, hemorrhagic events, and mortality risks [12]. Furthermore, there were no data on the characteristics of ICH, such as the etiology, size, location, and ICH score. Furthermore, the severity or stage of comorbidities was not adequately considered, even with the use of the CHA_2_DS_2_-VASc score for adjustments. Second, we conducted three independent 1:1 propensity score matching, which may cause problems with statistical validity. Third, we assessed severe thromboembolism, severe hemorrhage, and mortality as the endpoints, which for optimal accuracy, were defined by combining the ICD-10 codes, information on the emergency room visits, and information on the invasive process or vascular surgery undertaken. However, the fact that the theoretical definition of each event cannot exactly match the real event fundamentally limits this study, because have we used big data in health insurance. Fourth, our study did not exclude or identify patients with cerebral amyloid angiopathy or other contraindications to anticoagulants. This could have led to some prescriptions of antiplatelet agents or non-antithrombotic agents over anticoagulants, and may be a confounding factor in our analysis. Fifth, because we could not estimate the degree of disability of the patients who experience subsequently STE/SHE, we did not weigh up the comparative benefit of resuming anticoagulant therapy. Finally, because our results were based on a single ethnic population within 1 country with a relatively low incidence of hypertension, our results should be interpreted with caution.

In conclusion, this observational study on a nationwide cohort of patients with AF who survived an ICH and lived in an outpatient setting showed that resumption of anticoagulant was associated with a greater reduction in the STEs and SHEs as compared with resumption of antiplatelet usage or no treatment. Anticoagulation 6–8 weeks post-ICH showed a tendency of the lowest risk of STEs. Furthermore, NOACs were associated with reduced thrombotic events as compared with warfarin.

### CRediT authorship contribution statement

**Jong Youn Moon:** Investigation, Methodology, Writing – original draft. **Gi Hwan Bae:** Data curation, Formal analysis, Validation, Visualization, Writing – review & editing. **Jaehun Jung:** Conceptualization, Funding acquisition, Project administration, Writing – review & editing. **Dong Hoon Shin:** Conceptualization, Project administration, Resources, Writing – review & editing.

## Declaration of Competing Interest

The authors declare that they have no known competing financial interests or personal relationships that could have appeared to influence the work reported in this paper.
