# Supplementary material for: Restarting anticoagulant therapy after intracranial hemorrhage in patients with atrial fibrillation: A nationwide retrospective cohort study
Source: Int J Cardiol Heart Vasc. 2022 Apr 26;40:101037. doi: 10.1016/j.ijcha.2022.101037 (PMC9152296; doi:10.1016/j.ijcha.2022.101037)
Supplement: Supplementary data 1 [file mmc1.docx]

RESTARTING ANTICOAGULANT THERAPY AFTER INTRACRANIAL HEMORRHAGE IN PATIENTS WITH ATRIAL FIBRILLATION: A NATIONWIDE RETROSPECTIVE COHORT STUDY

**Supplemental material**

**Contents:**

**Supplementary Table 1.** International Classification of Diseases, Tenth Revision (ICD-10), codes for major events

**Supplementary Table 2.** Classification of antithrombotic prescriptions captured in the present study

**Supplementary Table 3.** International Classification of Diseases, Tenth Revision (ICD-10), mapping for major comorbidities

**Supplementary Table 4.** International Classification of Diseases, Tenth Revision (ICD-10), mapping for CHA_2_DS_2_-VASc score

**Supplementary Table 5.** Severe thrombotic events, or severe hemorrhagic events according to the use of anticoagulant and antiplatelet agents in patients with intracranial hemorrhage and atrial fibrillation after propensity score matching

**Supplementary Table 6.** Comparison of all-cause mortality, severe thromboembolic events, and severe hemorrhagic events according to time for resumption of anticoagulants

**Supplementary Table 7.** Comparison of beneficial effects on severe thrombotic events, and severe hemorrhagic events between NOACs and warfarin

**Supplementary Figure 1.** Flow diagram of inclusion of participants in the study

**Supplementary Table 1. International Classification of Diseases, Tenth Revision (ICD-10), codes for major events**

| **Diseases** | **ICD-10 codes** |
| --- | --- |
| **Severe thrombotic events** |  |
| Ischemic stroke | I63 |
| Other fatal thromboembolic events | I21, I22, I23, I74 |
| **Severe hemorrhagic events** |  |
| Recurrent intracerebral hemorrhage | I61 |
| Subarachnoid hemorrhage | I60 |
| Subdural hemorrhage | I62 |
| Epidural hemorrhage | G95.1 |
| Traumatic intracerebral hemorrhage | S06.360A |
| Esophageal hemorrhage | I850, I983 |
| Gastro-esophageal laceration hemorrhage syndrome | K22.6 |
| Ulcer with hemorrhage | K25, K26, K27, K28 |
| Unspecified gastrointestinal hemorrhage | K922 |
| Hemorrhoidal bleeding | I841, K858, I844 |
| Hemorrhage of anus and rectum | K625 |
| Hematemesis | K920 |
| Melena | K921 |
| Hemoperitoneum | K66.1 |
| Acute hemorrhagic gastritis | K29.0 |
| Hemarthrosis | M250 |
| Hemorrhage of prostate | N421 |
| Other uterine/vaginal bleeding | N93.8, N93.9 |
| Postmenopausal bleeding | N95.0 |
| Hemorrhage from throat/airway, unspecified | R048, R049 |
| Hemorrhage from throat | R04.1 |
| Hemoptysis | R04.2 |
| Epistaxis | R04.0 |
| Conjunctival hemorrhage | H11.3 |
| Choroidal hemorrhage | H31.3 |
| Retinal hemorrhage | H35.6 |
| Vitreous hemorrhage | H43.1 |
| Hemopericardium | I31.2 |
| Anemia following major bleeding | D629 |
| Hemorrhage not elsewhere classified | R58.9 |
| Otorrhagia | H92.2 |
| Hematoma of broad ligament | N83.7 |
| Postoperative hemorrhage | T810 |

**Supplementary Table 2. Classification of antithrombotic prescriptions captured in the present study**

| **Antithromobtics** |  |
| --- | --- |
| **Antiplatlet** | **Anticoagulant** |
| Clopidogrel | Warfarin |
| Aspirin | Rivaroxaban |
| Triflusal | Dabigatran |
| Dipyridamole | Apixaban |
| Cilostazol | Edoxaban |
| Prasugrel |  |
| Ticagrelor |  |

**Supplementary Table 3. International Classification of Diseases, Tenth Revision (ICD-10), mapping for major comorbidities**

| **Diseases** | **ICD-10 codes** |
| --- | --- |
| Heart failure | I50, I43, I099, I110, I130, I132, I420, I425, I426, I427, I428, I429 |
| History of venous thromboembolism | I26, I80, O87.1, O22.3, O22.5, I81, I82 |
| Ischemic heart disease | I65, I70, I71, I72, I73 |
| Valvular disease | I05, I06, I07, I08, I09.1, I34, I35, I36, I37, I38, I39, Z95.3, Z95.3, Z95.4, I342, I050, Q232 |
| Hypertension | I10, I11, I12, I13, I14, I15 |
| Diabetes mellitus | E10, E11, E12, E13, E14 |
| Dementia | F00, F01, F02, F03 |
| Hyperlipidemia | E78 |

**Supplementary Table 4. International Classification of Diseases, Tenth Revision (ICD-10), mapping for CHA_2_DS_2_-VASc score**

| **CHA2DS2-VASc** | **Score** | **ICD-10 codes or conditions** |
| --- | --- | --- |
| Congestive heart failure | 1 | I11.0, I50, I97.1 |
| Hypertension | 1 | I10, I11, I12, I13, I15 |
| Age≥75 | 2 |  |
| Diabetes mellitus | 1 | E10, E11, E12, E13, E14, |
| Stroke/TIA/thromboembolism | 2 | I63, I64, G45, I260, I269, I74 |
| Vascular disease: prior MI, peripheral artery disease, aortic plaque | 1 | I21, I22, I25.2, I70.0, I70.1, I70.2, I70.8, I70.9, I739 |
| Age: 65–74 | 1 |  |
| Female | 1 |  |
| Total score | 9 |  |

CHA_2_DS_2_-VASc: congestive heart failure, hypertension, age≥75 years, diabetes mellitus, stroke or transient ischemic attack, vascular disease, age 65–74 years, sex; TIA: transient ischemic attack; MI:Myocardial Infarction

**Supplementary Table 5.** Severe thrombotic events, or severe hemorrhagic events according to the use of anticoagulant and antiplatelet agents in patients with intracranial hemorrhage and atrial fibrillation after propensity score matching

|  | Anticoagulant users versus antiplatelet users  (Reference : Anticoagulant users) | | | Anticoagulant users versus non-users  (Reference : Non-users) | | | Antiplatelet users versus non-users  (Reference : Non-users) | | |
| --- | --- | --- | --- | --- | --- | --- | --- | --- | --- |
|  | HR | 95% CI | *P* | HR | 95% CI | *P* | HR | 95% CI | *P* |
| Severe thrombotic events | 1.168 | 0.930 – 1.465 | 0.181 | 0.385 | 0.312 – 0.475 | <0.0001 | 0.545 | 0.474 – 0.625 | <0.0001 |
| Severe hemorrhagic events | 1.013 | 0.845 – 1.214 | 0.889 | 0.578 | 0.487 – 0.685 | <0.0001 | 0.637 | 0.563 – 0.720 | <0.0001 |

CI: confidence interval; HR: hazard ratio

**Supplementary Table 6.** Comparison of all-cause mortality, severe thromboembolic events, and severe hemorrhagic events according to time for resumption of anticoagulants

|  | All-cause mortality | | | Severe thromboembolic events | | | Severe hemorrhagic events | | |
| --- | --- | --- | --- | --- | --- | --- | --- | --- | --- |
|  | HR | 95% CI | *P* | HR | 95% CI | *P* | HR | 95% CI | *P* |
| 0–2 : reference | 1.000 |  |  | 1.000 |  |  | 1.000 |  |  |
| 2–4 | 0.864 | 0.598-1.247 | 0.4341 | 0.799 | 0.557 - 1.145 | 0.222 | 0.620 | 0.462 - 0.830 | **0.001** |
| 4–6 | 1.15 | 0.809-1.636 | 0.4366 | 1.036 | 0.732 - 1.465 | 0.843 | 1.446 | 1.120 - 1.867 | **0.005** |
| 6–8 | 0.614 | 0.372-1.011 | **0.0552** | 0.588 | 0.362 - 0.957 | **0.033** | 0.864 | 0.614 - 1.217 | 0.404 |
| 8–10 | 0.616 | 0.338-1.123 | 0.1141 | 0.559 | 0.308 - 1.014 | 0.056 | 0.813 | 0.536 - 1.232 | 0.328 |
| 10–12 | 0.745 | 0.419-1.325 | 0.3164 | 0.722 | 0.408 - 1.279 | 0.264 | 1.172 | 0.790 - 1.739 | 0.429 |
| 12–14 | 0.808 | 0.388-1.685 | 0.5702 | 0.822 | 0.410 - 1.645 | 0.579 | 0.900 | 0.541 - 1.497 | 0.685 |
| ≥14 | 0.777 | 0.337-1.794 | 0.5547 | 0.687 | 0.298 - 1.583 | 0.379 | 1.173 | 0.675 - 2.039 | 0.571 |

All HRs are adjusted for gender, age and CHA2DS2-VASc(congestive heart failure, hypertension, age≥75 years, diabetes mellitus, stroke or transient ischemic attack, vascular disease, age 65–74 years, sex); CI: confidence interval; HR: hazard ratio.

**Supplementary Table 7.** Comparison of beneficial effects on severe thrombotic events, and severe hemorrhagic events between NOACs and warfarin

|  | Before matching  (Reference: NOAC) | | | After matching  (Reference: NOAC) | | |
| --- | --- | --- | --- | --- | --- | --- |
|  | HR | 95% CI | *P* | HR | 95% CI | *P* |
| Severe thrombotic events | 0.305 | 0.215 – 0.435 | <0.0001 | 0.263 | 0.144 – 0.480 | <0.0001 |
| Severe hemorrhagic events | 0.646 | 0.515 – 0.809 | 0.001 | 0.794 | 0.556 - 1.131 | 0.202 |

CI: confidence interval; HR: hazard ratio; NOAC: Novel direct anticoagulants

**
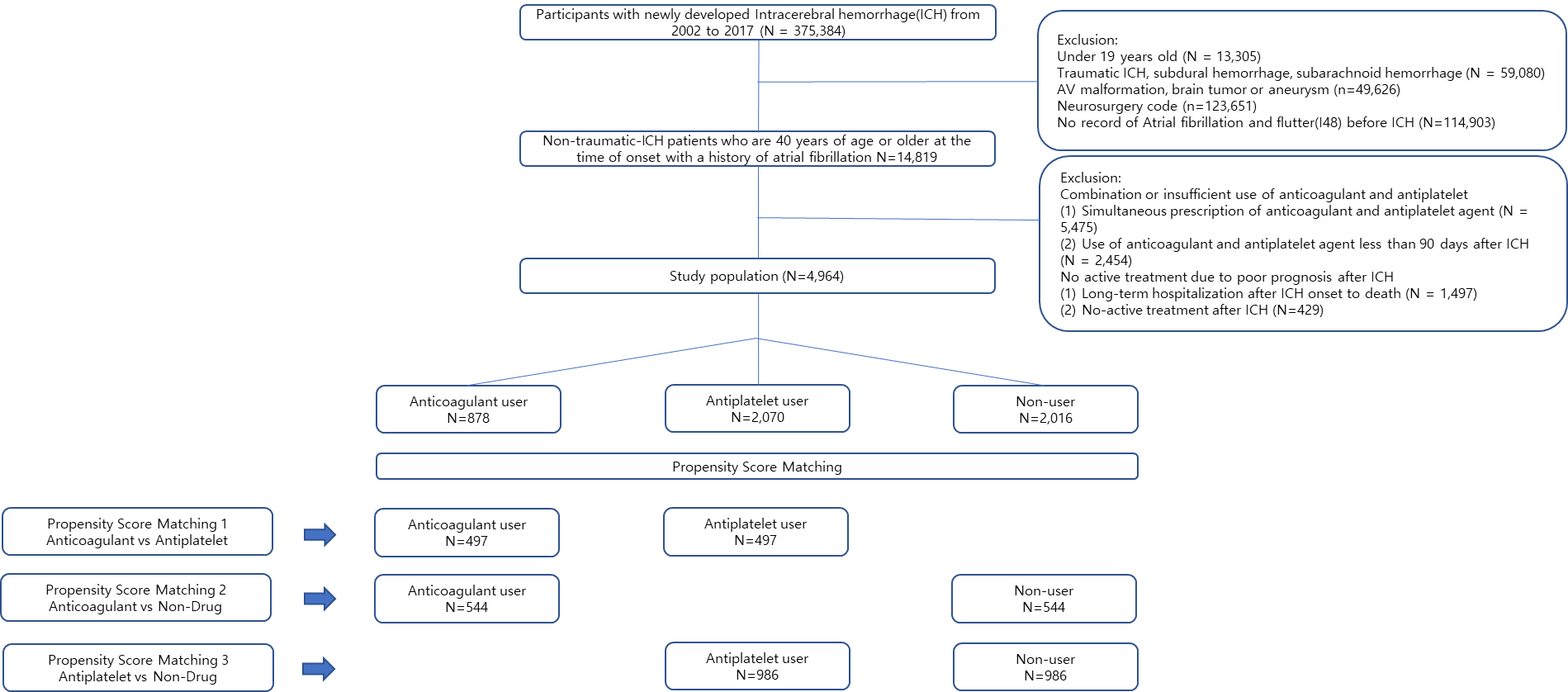
Supplementary Figure 1. Flow diagram of inclusion of participants in the study**
